# MPOInt: An Epigenetics-Driven Workflow for Integrative Multi-Level Proteomics

**DOI:** 10.1021/acs.jproteome.5c01076

**Published:** 2026-06-18

**Authors:** Zoe Schaefer, Carleigh Coffin Sokolik, Ivana K. Parker

**Affiliations:** † J. Crayton Pruitt Family Department of Biomedical Engineering, 130358 University of Florida, Gainesville, Florida 32610, United States; ‡ Emerging Pathogens Institute, 130358 University of Florida, Gainesville, Florida 32610, United States; § Faculty of African American Studies, University of Florida, Gainesville, Florida 32610, United States

**Keywords:** proteomics, epigenetics, epiproteome, phosphoproteomics, BCG, MCF-7, histone, disease enrichment, bladder cancer, breast cancer

## Abstract

Proteomics provides key insights into systems regulating cell behavior and can elucidate key epigenetic states influencing biological functions in health and disease. Integrating different modalities of proteomics (total, phospho, epi) allows for a comprehensive understanding of how systems change in response to a stimulus. A current limitation is that these data are often captured individually with a limited scope of how systems regulating biological processes interact. Integrative analyses are essential to accurately depict biological states but often require custom, time-consuming bioinformatics processing. The MPOInt pipeline and its associated R package provide highly flexible analysis modules that can be easily combined to develop a multidimensional view of the input data with minimal barrier to entry, and is capable of integrating histone proteome data with known epigenetic effectors. Using this tool and pre-existing clinical data, we identify several pathways that may be targets in driving the growth of aggressive cancers, including treatment-resistant estrogen receptor-positive breast cancer. Overall, MPOInt adds clinical context to data that is traditionally difficult to interpret and generates a unique network centered around epigenetic factors for each collection of data.

## Graphical abstract

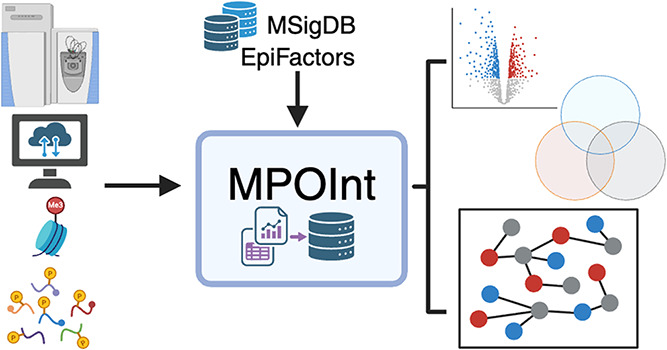

## Introduction

Proteomics has emerged as an essential tool in systems biology. New methods are being developed every year that increase the information able to be extracted from a single sample. Many of these methods focus on a single proteomic level, indicating a unique type of protein to be analyzed. Proteomics as an umbrella term describes the study of all proteins in a sample, which can vary in composition from cell culture lysate,^1,2^ to biological fluids such as urine^3,4^ or blood,^5–7^ to tissue biopsies^8–10^ from patients. Emerging technologies allow for absolute and relative quantification of targeted peptides in each sample.^11–13^

Proteomics enables the identification and targeting of proteins based on unique properties, such as post-translational modifications (PTMs) or cellular location and function. This approach facilitates the targeted identification of specific protein subsets within a sample that possess distinct regulatory functions. For example, phosphoproteomics identifies phosphorylated proteins that are enriched by leveraging properties of negatively charged phosphate groups.^14,15^ Common methods include immobilized metal affinity chromatography (IMAC) or titanium dioxide columns, which use positively charged metal ions to bind phosphate groups.^16,17^ Phosphorylated proteins can then be eluted separately from the rest of the sample. Similar techniques exist for other post-translational modifications (PTMs), including acetylation and acetylomics^18,19^ as well as ubiquitination and ubiquitinomics.^20,21^ Cellular compartments and protein types also have distinct proteomic subsets: exosome proteomics looks at extracellular vesicles,^22,23^ while epiproteomics examines histones and their associated PTMs.^24^ While techniques for obtaining this information continue to evolve,^25^ few tools exist to connect each proteomic level.

Proteomics data is inherently large-scale, identifying thousands of peptides per scan. Databases such as the PRIDE Archive store tens of thousands of data sets, with the PRIDE Archive approaching 285 petabytes of data as of 2024.^26^ Much of this untargeted proteomics and phosphoproteomics data, collected to answer unique biological questions, contains an abundance of untapped information that can provide deeper insight into disease states and normal physiology.

However, there is a notable lack of accessible tools to perform integrative analyses of data, connecting the proteome and phosphoproteome to highlight novel regulatory networks.

Exploring the epigenetic landscape using proteomics has improved our understanding of the many PTMs and regulatory factors that influence a cell at any given time.^27^ Collected data can be applied to discover new epigenetic regulatory networks. Systems-level analysis offers valuable insights, as a single protein may be influenced by numerous overlapping regulatory pathways. Integrating data from the total proteome, phosphoproteome, and histone proteome helps identify and discover these interactions. All cellular activity is driven at some level by epigenetics, primarily regulated through altered gene accessibility, commonly by changing DNA methylation or histone modifications.^28^ Examining the total proteome alone shows relative quantities of a protein, but does not indicate how that protein is being modified, its activity, or its transcription levels, among other important regulators of cellular states. However, this problem cannot be solved by looking at individual proteomics data sets in isolation.^29^

Despite growing knowledge of epigenetic effectors—proteins and complexes that write, edit, and erase epigenetic marks—major gaps remain in understanding how they regulate target genes and respond to cellular stimuli.^28^ Analyzing proteomics data in multiple levels provides a unique way to fill this gap. Current studies are limited by either the lack of epigenetic-focused analytical tools for mass spectrometry data or the narrow scope of targeted methods like Western blotting.^30^ These approaches often examine only one layer of regulation, and present a major opportunity to further explore the complexity of interconnected cellular pathways.

To address this issue, we have created MPOInt (Multilevel ProteOmics Integration), an updated multilevel proteomics approach focusing on identifying mediators of epigenetic regulation from total, phospho, and histone focused proteomics data. It is a bioinformatics pipeline developed in R and visualized interactively in Python, intended to allow users to accelerate discovery, enable clinical translation, and quickly adapt to additional proteomics applications with minimal computational resources or computer science expertise required. Proteins meeting user-defined thresholds for fold-change and statistical significance, are assembled to create a framework of histones and associated effectors, providing a unique epigenetics-centered view of proteomics data. Moreover, we demonstrate how users can easily use existing data sets to elucidate hidden epigenetic regulatory networks. The data output here, with additional visualizations, is available in Markdown workbooks that allow users to recreate the figures included in this publication. These files can be downloaded along with the pipeline package at github.com/zoe-schaefer/MPOInt.

## Methods

### Study Cohorts and Sample Preparation

To develop and validate this pipeline, two collections of data were used. The first set consists of total and histone proteome data examining THP-1 macrophages after BCG infection as published by our group (ProteomeXchange accession PXD051187).^31^ This cohort also includes the accompanying phosphoproteome data (PRIDE Database accession PXD013171), initially used to examine differences between BCG and virulent tuberculosis infection in THP-1 macrophages.^32^ Sample preparation protocols are described in the respective publications. The resulting total and phosphoproteome data were processed for peptide identification in Proteome Discoverer, then exported to a.xlsx file for downstream analysis. The histone proteome data was interpreted with EpiProfile in MATLAB,^33^ which returns a.csv file along with spectrograms for all identified histone peptides.

Another data set was used to ensure input flexibility in an alternate disease state. A study on stemness behavior and matrix stiffness using MCF-7 cells was selected due to public availability of proteomic, phosphoproteomic, and epigenetic data. Each level of data for these cells had a different output format than the first case, and were processed in Proteome Discoverer (total and phosphoproteome) and EpiProfile (histone proteome). MCF-7 data was processed with MaxQuant and Perseus (total and phosphoproteome), as well as a pre-EpiProfile custom MATLAB script (histone proteome).

#### Total/Phosphoproteome

The total and phosphoproteome data were retrieved from a study examining 3D culture matrix stiffness and its correlation with MCF-7 stemness and resistance to dormancy.^34^ Data-dependent analysis (DDA) was performed in MaxQuant^35^ (v1.5.2.8) and Perseus^36^ (v1.6.5.0), then exported to.xlsx format and retrieved from the ProteomeXchange database with identifier PXD046799. Of the matrices of varying stiffness studied, we selected the 90 Pascal matrix (marked M90 in data) due to its association with aggressive tumor characteristics such as high stemness and proliferation. In this analysis, the M90 data was compared to the flat surface (F in data), which serves as a control for the typical culture environment.

#### Histone Proteome

Histone proteome data was previously collected as a panel on various cancerous and noncancerous immortalized cell lines^37^ in 2D by Garcia et al. This data was originally uploaded to the Tranche database, but raw files are now available on MassIVE with file IDs MSV000078413, MSV000078403, MSV000078401, and MSV000078400. These can also be found by searching the source Tranche hashes in the MassIVE database. For ease of analysis, we used the normalized abundances provided as a supplementary Excel sheet in the original publication. For the purposes of this pipeline, we examine characteristics that differentiate MCF-7 cells from noncancer cells by using the noncancerous cell lines (HEK-293, HaCaT, hESC2, HFF, Mdm13) as collective controls. We utilize this data for the histone component of our pipeline, due to the fact that all levels of proteome data are not included in the previous publication. For each identified histone fragment, all control cell lines were averaged to generate a baseline to which the MCF-7 fragment abundances were compared. Original MS data analysis was initially performed by in-house software, then validated by manual inspection, and exported. This abundance data was exported to.xlsx and used in the current study.

### MPOInt Package Development

The MPOInt pipeline was developed to address challenges in integrating multilevel proteomics data, including the lack of tools that focus on epigenetics. The primary output of this package is the interactive network visualization, which is constructed by combining separate analyses of total, phospho, and histone proteomics data. To create this network and develop the pipeline, several goals and core functions were identified. The package should enable robust analysis of individual proteomics and epiproteomics data sets, support disease enrichment analyses for target discovery, and provide intuitive network visualizations with exportable analytical outputs. These functions must also be possible with minimal user adjustments across different data sets and input formats.

#### Data Processing

To eliminate the need for proprietary mass spectrometry software, this pipeline is designed to accept files with identified and quantified peptides in tabular or spreadsheet formats such as.csv and.xls files. Two main formats of data are addressed here: data with summarized abundances (mean and standard deviation) for each condition and data with values for individual samples. Unified protein annotations are assigned with OrganismDbi^38^ (v1.48.0). Users can set thresholds for fold change and statistical significance, as well as lists of proteins to highlight in initial visualizations if potential targets are known. These are displayed in volcano plots as the first figures generated.

For epiproteome data, processing of the input file is required due to the input format. The data produced by programs like EpiProfile can be difficult to read, so a portion of the import function is dedicated to making values interpretable. Calculations for fold change and *P*-value are not performed by EpiProfile, so we include a basic *t* test to generate a *P*-value and manually obtain a fold change value between two conditions as identified by the user. Some epiproteome data is present only as mean values and standard deviation, so settings can be adjusted to use a T score to calculate a *P*-value in these cases.

#### Pathway Enrichment

Enrichment for GO and KEGG pathways is performed with clusterProfiler^39^ (v4.12.6) on both the proteome and phosphoproteome data. GO pathways are broken down into their three separate subcategories for upregulated and downregulated proteins. Dot plots are generated to show the raw gene count in each pathway, as well as a relative gene count comparing the raw count to the size of the overall pathway so users can quickly identify highly influential changes.

#### Disease Enrichment

MSigDB (v2025.1.Hs)^40,41^ is a collection of annotated gene sets, many of which are linked to disease ontology terms. The user selects a data set to use, then with the MSigDBR R package (v25.1.1)^42^ we retrieve that gene list. We compare the “hits” (proteins above the fold change and *p*-value thresholds) from the proteome and phosphoproteome data sets, and return both a visualization of the overlap (ggvenn v0.1.10 ^43^) and a list of the proteins in each intersection.

#### Epigenetic Enrichment

EpiFactors is a curated database of epigenetic factors, targets, and products, containing over 1000 interacting proteins, lncRNAs, and genes.^44^ This is used as a central structure to assign proteins to known complexes. This also allows for assignment of targets and resulting PTMs associated with each complex. The user points the file to a downloaded form of this data, which then matches proteins from all three levels of the proteome.

#### Network Generation and Visualization

Proteins are included in the network if they have a hit in the proteome or phosphoproteome, as well as a connected hit in the epiproteome through their associated complex. Multiple visualizations are generated, including an interactive network with a short Python call to extend graphics capacity beyond that of R alone. The network itself is multidimensional, so parameters are provided for the user to customize their view and export the network as a static image.

### Statistical Analysis

If abundance data for each sample is available, an additional section can be run to identify proteins only expressed in one condition. Statistical analysis is directly performed on the raw data in the R script, which calculates unadjusted *P*-values for the histone proteome using a two-sided *t* test. This section can be copied and used for the total proteome and phosphoproteome sections if *P*-values are not included in the data export.

If data for individual samples is not available, but group means and standard deviations are, the Markdown file analyzing MCF-7 cells demonstrates how a T-score and *P*-value can be calculated manually. Here, we demonstrate use of both data with aggregated mean values with standard deviations (MCF-7 epiproteome as provided in an atlas) and abundance values for individual samples, expanding the application to data sets which do not have the full raw files available.

Adjustment for multiple testing is performed in the pipeline. For proteome and phosphoproteome data, Storey’s *q*-value is reported to control false discovery rate (FDR) in data sets with very high feature counts.^45^ The epiproteome data can be adjusted with the Benjamini-Hochberg correction, and unadjusted *P* values are also provided to allow for flexibility at the user’s discretion considering the smaller size of these experiments.

### MPOInt Pipeline Validation

To validate the individual proteomics processing, we recreate analyses performed in a previous publication to ensure result accuracy. The “BCG case” Markdown file on GitHub contains the full pipeline output for this data, and selected figures are discussed here. We then used the second cohort of data to perform a second analysis, which is presented in full in the “MCF7 case” example document. The analyses performed in this publication can be entirely recreated with the data included in the R package. This provides working examples that users can modify or reference when analyzing other data.

For the disease enrichment portion, we selected three areas of clinical interest for the two experimental data sets. MSigDB contains multiple unique pathway sources. Pathway identifiers beginning with WP are sourced from WikiPathways,^46^ those beginning with HP: are from the Human Phenotype Ontology database,^47^ and those beginning with R-HSA are from Reactome.^48^ We also include one primary source from a gene expression study in the Curated subcollection in MSigDB.^49^ For BCG-infected THP-1 cells, we examined genes connected to bladder cancer (WP2828), melanoma (HP:0002861) and RSV infection (R-HSA-9833110). For the MCF-7 data, we focused on the condition most associated with high stemness (90 Pascal matrix), and selected genes upregulated in early estrogen receptor-positive breast cancer (M18299), associated with hypertension (HP:0000822), and enriched in ovarian cancer (HP:0100615).

### MPOInt Use

To use this pipeline, users should have three data sets to compare. In this publication, we use total proteome and phosphoproteome data, but these can be replaced with other proteomic data types such as acetylome or ubiquinome as no PTM-specific operations are performed. Histone proteome data is intended for use with EpiProfile output, but is not restricted to that format. This package can be used with PTM types not currently supported by EpiProfile, as processing is limited to text parsing. If non-EpiProfile data is used, users can skip the data cleaning step included here and format the data prior to pipeline use.

Each data set used in this pipeline should have been processed for peptide identification after mass spectrometry. The minimum features required in a proteomics data set are the following: (1) A protein identification symbol–UniProt, ENTREZ ID, or HGNC Symbol among others can be mapped to common gene symbols with accessory tools; (2) A calculated log2 (Fold Change) for the two conditions being compared; and (3) A *P*-value or *q*-value, either unadjusted or corrected. For data sets without this information, analysis may still be possible (see analysis of the MCF-7 epiproteome data) if means and statistical descriptions are available.

## Results and Discussion

### The MPOInt Pipeline Generates Accurate Visualizations of Multilevel Proteomics Data

This pipeline is intended to facilitate both rapid discovery of epigenetic networks and provide an in-depth analysis of multilevel proteomics data.

BCG (bacille Calmette-Guerin) is a strain of *Mycobacterium bovis* used globally to vaccinate against tuberculosis and is a known activator of trained immunity in macrophages.^50^ It is also a common treatment for nonmuscle-invasive bladder cancer.^51^ Previously, we published a study of epiproteomic, proteomic, and phosphoproteomic data which revealed novel epigenetic pathways in a model of BCG infection.^31^ Manual integration of these three data sets generated a small network limited to acetylation, where NuA4, NuRD, NSL, Sin3A, SIRT3, and SIRT6 were identified as potential epigenetic effectors. This analysis highlighted pathways that remain understudied in trained immunity and other BCG use cases. However, its practicality was limited due to the significant time required, potential for error, and difficulty of managing data sets of this scale by hand. To demonstrate the capacity of this pipeline, we used the same data as a validation case to ensure its reliability and perform analyses deeper than those conducted previously.

This high-level overview of BCG infection in THP-1 macrophages is a collection of figures (Figure 1) generated by the pipeline with its default parameters, and accurately depicts data highlighing standout “hits” (peptides meeting both the thresholds for fold change and statistical significance) in all three levels of the proteome, as well as a subset of the enriched pathways. In this case, we use a fold change threshold of √2 and a *P*- or *q*-value of 0.05 for the epiproteome or total and phosphoproteomes, respectively. We present these as a demonstration of the pipeline’s ability to quickly produce visualizations for exploratory analysis of individual proteome levels before beginning the integrated analysis.

**Figure 1:**
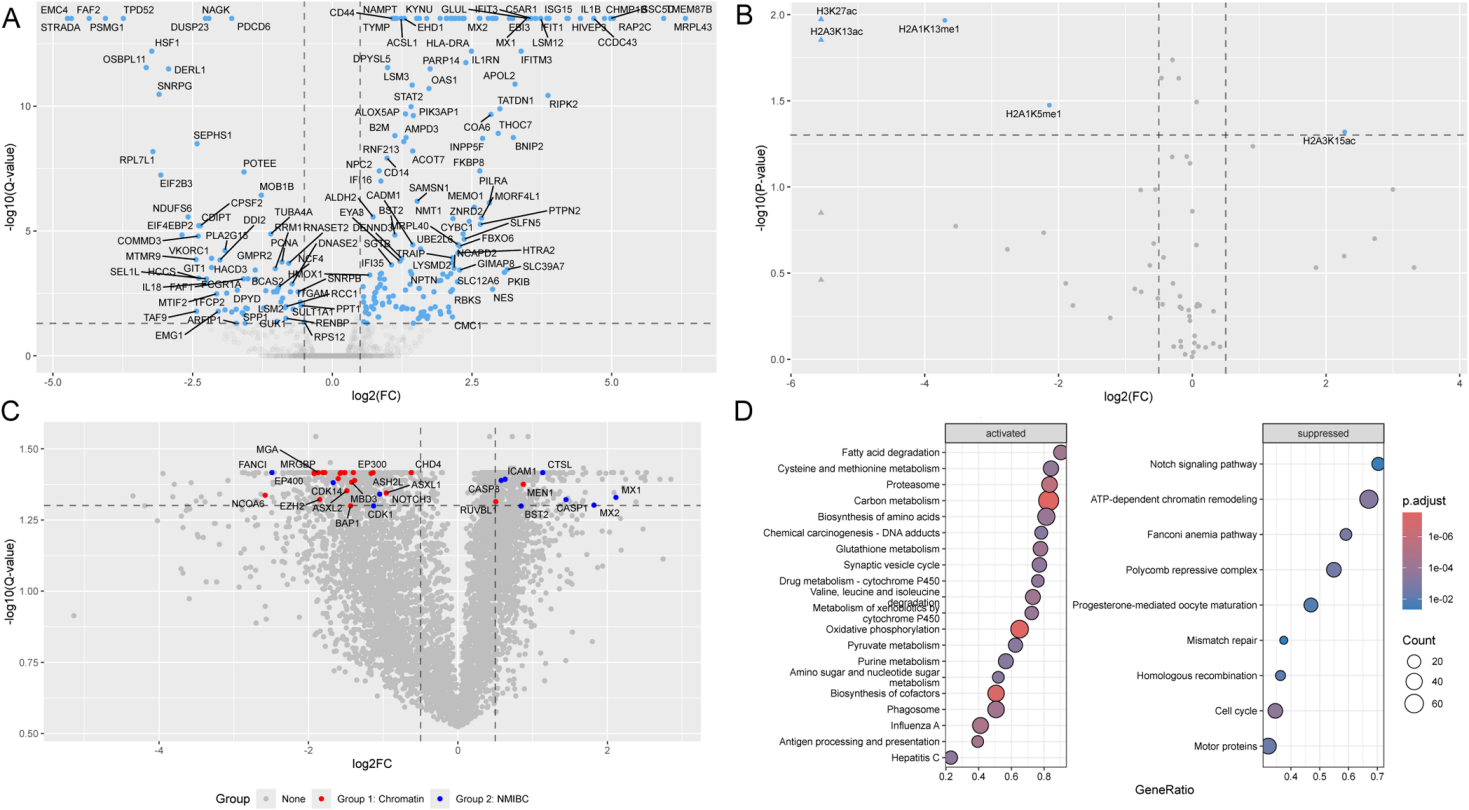
Selected output figures from the MPOInt pipeline using BCG infection. (A) Total proteome; (B) Histone proteome; (C) Phosphoproteome (highlighted hits of interest); Group 1 - hits from known chromatin modifiers. Group 2 - targets of interest in bladder cancer; (D) Top 20 differentially enriched KEGG terms in the phosphoproteome. (A–C) The *X* axis represents the log2 transformed fold change for infected vs control cells and the *Y* axis represents the −log10 transformed *P* or *q*-value. (D) The *X* axis shows the ratio of genes in a total pathway present compared to the full pathway size.

Figure 1 depicts the data visualization possible with this pipeline. Overviews of the total proteome and histone proteome can be generated (Figure 1A,B), and if genes of interest are known, highlights can be added to those hits as shown in the phosphoproteome (Figure 1C). Figure 1D displays one of multiple gene set enrichment plots, which provides insights into key pathways being influenced by a treatment under investigation or cellular states enhanced after bacterial stimulation.

BCG infection of macrophages has been associated with key changes in cellular behavior, primarily associated with secretion of pro-inflammatory cytokines, metabolic shifts, and epigenetic reprogramming. Intracellular signaling cascades initially result in increased secretion of TNF-α, IL-1β, IL-6, IL-12, and IFN-γ.^50,52–54^

Following the initial response, the signaling cascades promote glycolysis rather than oxidative phosphorylation.^55^ The resulting increased metabolic changes promote epigenetic modifications. As identified by targeted studies of trained immunity, this causes accumulation of H3K27ac at active enhancers and active promoters, H3K4me1 at poised enhancers, and H3K4me3 at active promoters.^52,56,57^

As a direct result, long-term trained immunity develops. This phenotype is associated with enhanced response to nonspecific stimuli (shorter response time to peak and larger amplitude) including viral and fungal pathogens, increased pattern recognition receptor and MHC molecule expression, and lasting changes in lipid metabolism and glycolytic pathways.^52,57–61^

As in the previous work, we find differential expression of notable proteome hits in infection and trained immunity, including upregulation of IL-1β, IFIT1, IFITM3, and IL1RN. All of these proteins are linked to infection response, with IL-1β being of particular interest in the development of trained immunity.^62^ IFIT1 and IFITM3 are antiviral proteins involved in viral entry and RNA recognition, and IL1RN encodes the essential IL-1 receptor antagonist IL-1Ra. Also consistent with prior analysis, multiple epigenetic effectors are differentially regulated in the phosphoproteome, including EZH2, RING1, and EP300.

Finally, pathway analysis highlights cellular activities after BCG infection, including immune response regulation, phagocytosis, and MHC receptor binding. These findings are in line with previous studies of BCG-infected macrophages,^52,63^ indicating that the pipeline successfully processes data without affecting hits.

### Disease Enrichment Reveals Potential Mechanisms Behind BCG’s Effects in Clinical Applications

As part of the pipeline, users can compare their data with data sets from MSigDB. These lists contain curated databases of genes associated with a disease of interest, as well as evidence and publications on the subject. To demonstrate how this can be used for discovery, we generated three Venn diagrams that compare the hits from the total proteome and phosphoproteome with the GDA lists for three areas of interest in BCG research. BCG is already used as a first-line treatment in a subtype of bladder cancer (Figure 2A, WP2828),^64^ and is being used in clinical trials as an adjuvant for melanoma treatments (Figure 2B, HP:0002861).^65^ Overlaps in these regions could indicate potential mechanisms for the efficacy of BCG as a therapeutic agent. In the bladder cancer segment, the sole member of the center section is TYMP, a thymidine phosphorylase. This molecule is overexpressed in many cancers, and in bladder cancer is closely linked to poor prognosis, advanced tumor staging, and resistance to chemotherapy treatment. This gene was upregulated in other studies of BCG treatment in the bladder cancer environment, but has not yet been investigated in this context. This tool therefore could indicate a potential direction for further research into mechanisms of BCG treatment in bladder cancer.

**Figure 2:**
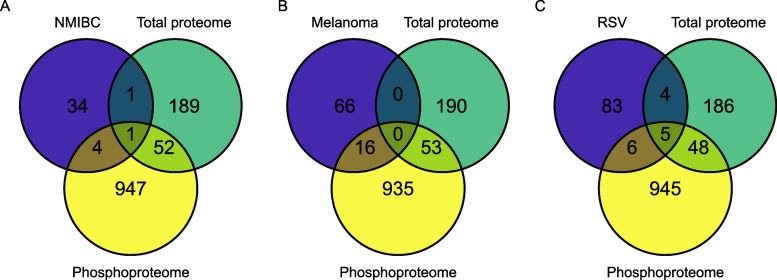
Disease enrichment from 3 areas of investigation for BCG use. (A) Enrichment with nonmuscle-invasive bladder cancer (NMIBC); (B) Enrichment with melanoma; (C) Enrichment with respiratory syncytial virus (RSV). Purple represents diseases of interest, blue represents the total proteome, and yellow represents the phosphoproteome. Numbers in each section indicate the number of proteins in a category.

Finally, BCG is known to have cross-protective effects in trained immunity,^66^ and was previously investigated as a preventative measure in COVID-19 infection^67^ as well as other respiratory infections, including respiratory syncytial virus (RSV, R-HSA-9833110).^68^ RSV is a common viral pathogen that can be particularly severe in infants^69^ (Figure 2C). One of the proposed mechanisms for BCG-induced viral defense is through activation of interferon-mediated pathways.^62^ At the center of the Venn diagram in Figure 2C are five hits, all of which are directly involved in interferon response and signaling. Two cytosolic double-stranded RNA (dsRNA) sensors are present, RIG-I and IFIH1 (MDA5). When activated, these start a cascade resulting in the cell generating type I interferons such as IFN-α and IFN-β.^70^ The antiviral response can also be initiated by EIF2AK2, an autophosphorylating protein kinase that is activated by dsRNA and halts protein translation.^71^ The ongoing cellular response is orchestrated in part by ISGylation, where ubiquitin-like proteins are ligated onto effector molecules to change their behavior in response to interferon signaling.^72^ Here, we find altered expression of ISG15 and UBE2L6, a common ISGylating protein and its associated conjugating enzyme.^73^ The identified hits span multiple antiviral pathways, many of which are specific to dsRNA viruses. This may indicate a reason for the observed antiviral activity of BCG. These effectors notably all have evasion mechanisms associated with RSV–for example, the RSV N protein directly reduces dsRNA recognition.^74^ It is possible that some viruses are able to continually evade BCG-induced antiviral processes or that BCG does not upregulate pathways targeting more diverse viruses, which would support the mixed evidence in its efficacy as a general antiviral vaccine.^75^ The MPOInt pipeline therefore allows for discovery of pathways across a wide range of topics as a clinical research tool to streamline and highlight hits in conditions and treatments of interest.

The pipeline also outputs a list of the overlapping hits. For brevity, we only include the NMIBC overlap here; hits for melanoma and RSV are available in Supporting File 1. Table 1 is generated by the MPOInt pipeline, allowing researchers to quickly identify targets of interest.

**Table 1:** Hits Identified in the Disease Enrichment Section of the Pipeline for the NMIBC Genes of Interest

intersection	hits
NMIBC/Phosphoproteome	BRAF, NRAS, RAF1, RB1, TYMP
NMIBC/Total proteome	MMP9, TYMP
Total proteome/Phosphoproteome	ACSL1, ALDH2, CMPK2, CNP, DDI2, DERL1, EIF2AK2, EYA3, FKBP8, FTL, GBP1, HELZ2, HMBS, HMOX1, ICAM1, IFI16, IFI44, IFIH1, IFIT1, IFIT2, IFIT3, INPP5F, ISG15, LSM3, MOB1B, MX1, MX2, NAGK, OAS1, OAS3, OASL, PARP14, PKIB, RIGI, RNF213, RRM1, RSAD2, SAMD9, SIRPA, SIRT2, SLFN5, SNRPG, SRSF10, STAT1, STRADA, SULT1A1, TAP2, TAPBP, TUBA1A, TYMP, UBE2D3, UBE2L6, VKORC1
NMIBC/Total proteome/Phosphoproteome	TYMP

### MPOInt Creates a Clear Network Connecting Clinical Applications of BCG to Three Levels of the Proteome

One of the defining features of this pipeline is the integrated network visualization for epigenetic marks. The network generated for BCG-infected THP-1 macrophages is shown in Figure 3, only including proteins with known complex associations. Since one protein or PTM can be associated with multiple complexes, we include a chart view (Figure 3A) that shows the complex members (*Y*-axis) and complexes (*X*-axis), colored by histone PTM. Figure 3B shows a static version of the interactive network visualization, where gray points were identified from the histone proteomics data and blue points were identified in the phosphoproteome. Triangles indicate relative expression in infected cells. Additionally, multiple tables are generated to clearly describe the results. Table S3 presents the PTMs and complexes associated with each protein in the network, as well as the log2-transformed fold change of the protein and its source. Table S4 similarly presents this information for each PTM, with the fold change from the epiproteome included.

**Figure 3:**
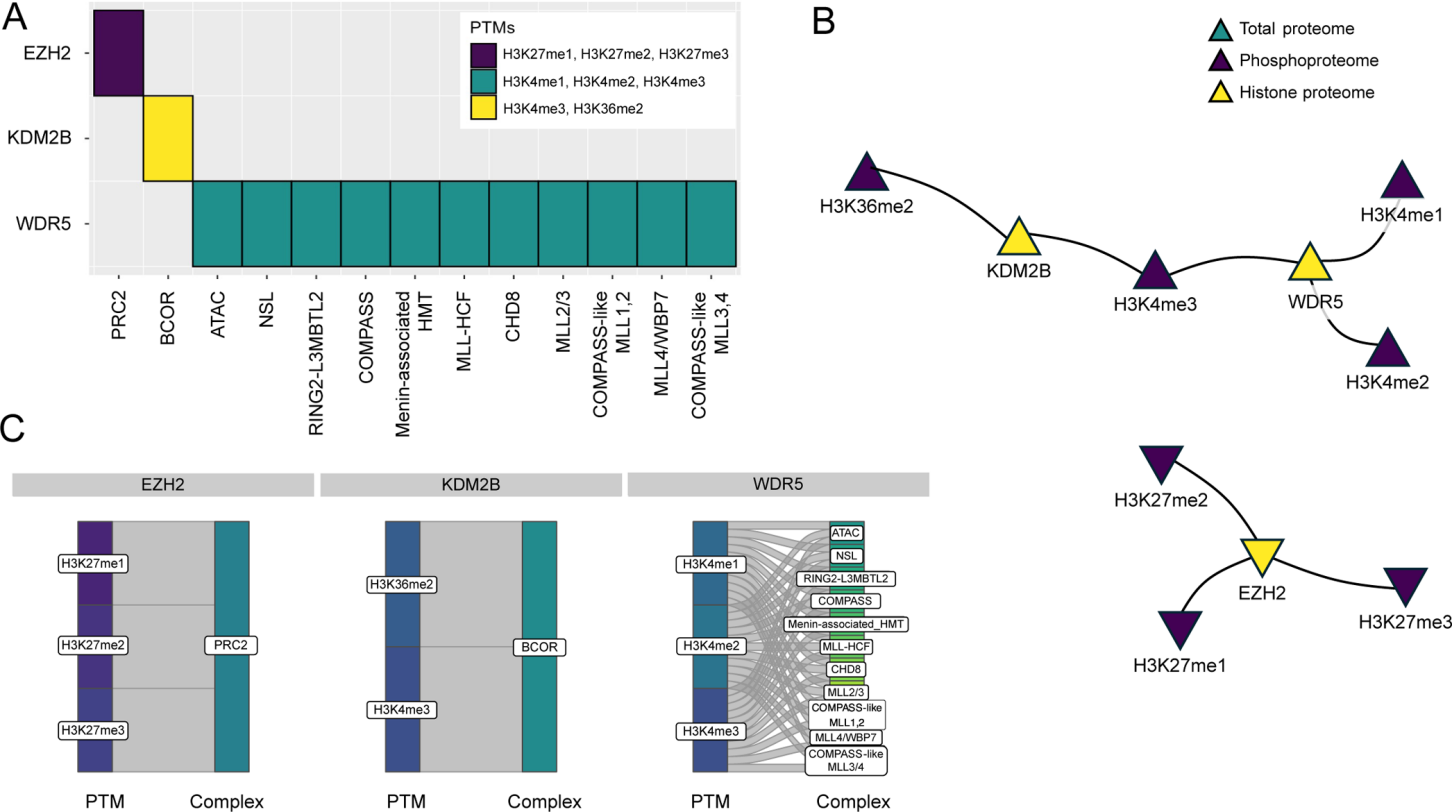
Epigenetic networks from BCG studies. (A) A graphical representation of each complex (*x*-axis), PTM (Color coded bars), and complex member identified in the network (*y*-axis); (B) Histone PTMs assessed in the histone proteome (purple triangles) connected to phosphoproteome (yellow triangles) regulators. The direction of each triangle indicates whether a protein was upregulated or downregulated in the condition of interest; (C) Sankey diagrams showing the connections between PTMs and complexes for each effector.

With this method, we can identify two main connected subnetworks. At the top of Figure 3B, we see that EZH2 is connected to H3K27me1, H3K27me2, and H3K27me3, all of which are downregulated. Figure 3A shows that this connection is made through the PRC2 complex, which is an essential mediator of gene repression as it catalyzes H3K27 mono-, di-, and trimethylation through its catalytic subunit, EZH2. Both gain-of-function and loss-of-function mutations in PRC2 members in somatic cells often result in tumorigenesis, and targeted therapies continue to emerge as treatment options for these cancers. In NMIBC, high activity of EZH2 has been correlated with poor prognosis and rapid tumor progression. Since BCG infection reduced EZH2 phosphorylation and the presence of methylated H3K27, this could indicate a mechanism for the efficacy of BCG in treating NMIBC.

The second network in Figure 3B is centered around WDR5 and KDM2B. KDM2B is associated with the BCOR complex (also called PRC1.1), where it localizes to nonmethylated DNA in CpG-rich promoters. In this complex, KDM2B functions as a histone reader rather than a demethylase. Both the canonical PRC1 and the noncanonical PRC1.1 use a ubiquitin ligase subunit to generate H2AK119 monoubiquitination. PRC1 is dependent on H3K27me3 for its targeting mechanism, but PRC1.1 is recruited independently of H3K27me3. This difference allows PRC1.1 to rapidly localize to active promoters in response to cellular stress, making it an effective tumor suppressor.

WDR5 reads methylated H3, and several complexes use it as a scaffold to assemble and perform their functions on the histone. The primary complex that overlaps with PRC1.1 is COMPASS, which uses WDR5 to localize to active promoters. COMPASS recruitment results in methylated H3K4, a classical marker of gene transcription.

Co-occurrence of H3K4 methylation and H2AK119ub1 marks indicate a form of poised gene or bivalent promoter. Ubiquitin-dependent poised genes use H2AK119ub1 instead of H3K27me3. Compared to a traditional H3K27me3-dependent poised gene, ubiquitin-dependent poised genes have been associated with a faster response and more rapid “switch” behavior following stimulation. Given that EpiProfile cannot identify H2AK119ub1 without specific preparation methods to preserve ubiquitination, we suggest that further work should be conducted to determine the role of ubiquitin-dependent poised genes in trained immunity and the time dynamics of the associated secondary response.

Here, we use phosphoproteome data as a general measure of change in the activity or presence of a molecule in the two conditions being compared. Because this tool is intended to identify overall changes in cellular states, reducing separate phosphorylation sites into a single indicator for each molecule allows us to reduce overall noise. Additionally, not all phosphorylations have clear correlations to protein activity or conformation. The shifts identified in this network view can be referenced in the original phosphoproteome data to identify the residue phosphorylated, and should be explored in detail with targeted follow-up studies.

### MPOInt Enables Discovery Proteomics in MCF-7 Cells

To demonstrate that the MPOInt pipeline is applicable across multiple data sets, we analyzed publicly available multilevel proteomics data from MCF-7 cells, a cell line derived from estrogen-responsive breast cancer. Here, MCF-7 cells were grown in 3D culture matrices of varying stiffnesses. We selected to compare the 90 Pa matrix compared to the flat control surface since this condition was associated with high stemness and cell proliferation^34^ (Figure 4). Again, here we use a fold change threshold of √2 and a *P*- or *q*-value of 0.05 for the epiproteome or total and phosphoproteomes.

**Figure 4:**
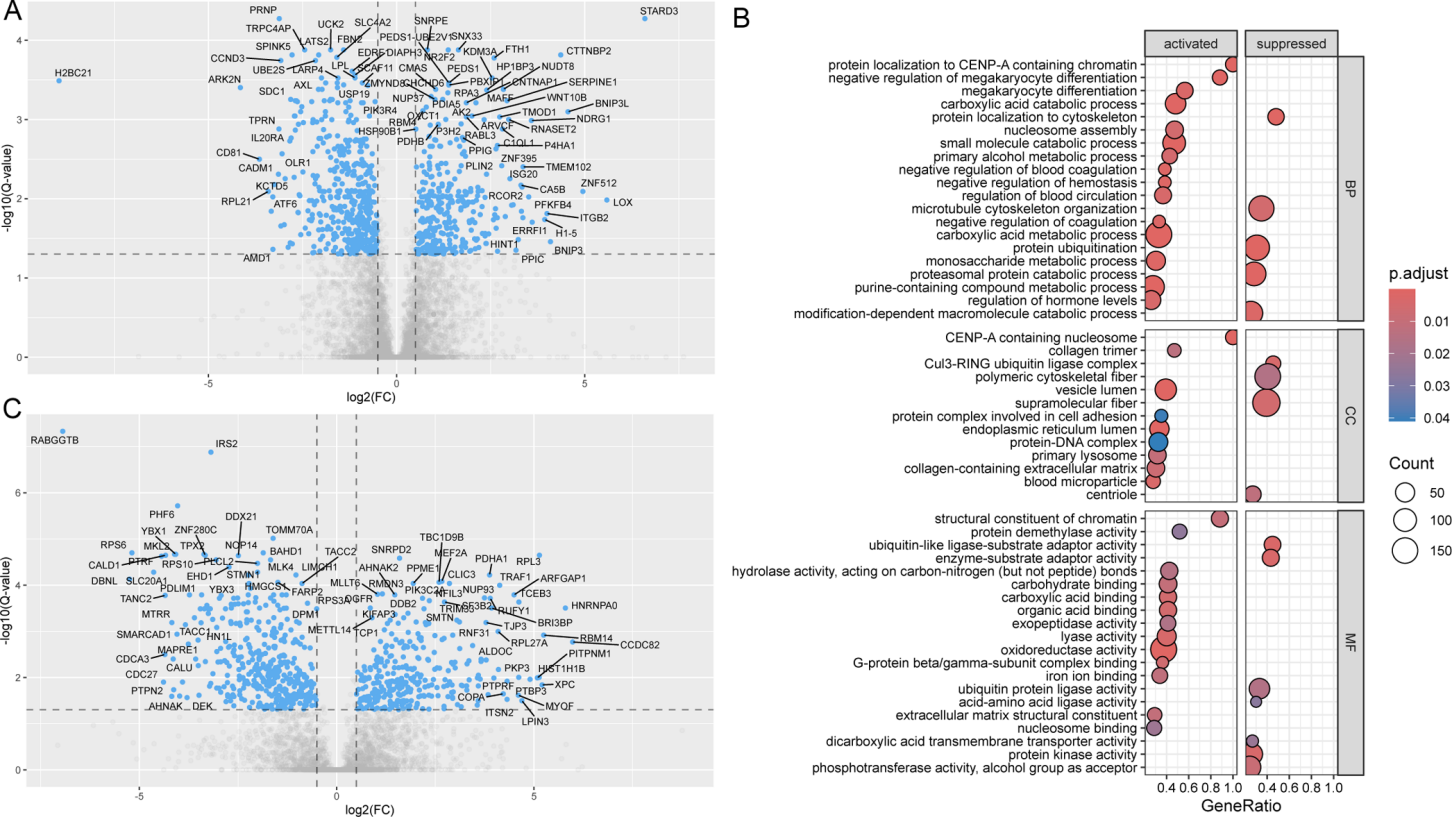
Proteome and phosphoproteome figures from the pipeline using MCF-7 stemness data. (A) Total proteome; (B) 20 upregulated pathways from each GO category in the proteome; (C) Phosphoproteome; (A, C) The *X* axis represents the log2 transformed fold change for 3D-cultured vs control cells and the *Y* axis represents the −log10 transformed *q*-value. (B) The *X* axis shows the ratio of genes in a total pathway present compared to the full pathway size.

In Figure 4A, we show another overview of the total proteome and its pathways. Using these overviews, we can quickly identify proteins and pathways of interest in the condition being investigated. Proteins heavily implicated in cancer physiology such as STARD3 ^76^ and NR2F2 ^77^ are upregulated, along with pathways indicating significant shifts in cellular metabolism. The corresponding phosphoproteome data is displayed in Figure 4C. A highlight of these panels is the upregulation of phosphorylated forms of multiple ribosomal proteins, including RPL3 and RPL27A. This is reflected in the gene set enrichment (Figure 4B) which shows a high number of RNA processing pathways. These findings confirm that the MPOInt pipeline is able to produce and interpret epigenetic targets in pre-existing multilevel proteome data.

### MPOInt Interprets and Presents Meaningful Epiproteome Data in Breast Cancer Stemness

Given that epiproteomics data is not easily interpreted in proteomics processing software, we set out to create reusable code that can parse this data as output from either EpiProfile 2.0 (as in Figure 1B) or other custom processing scripts with similar naming conventions, as shown in Figure 5. We provide a view of co-occurring PTMs as interpreted by mass spectrometry (Figure 5A) as well as a summary of individual PTMs and unmodified residues (Figure 5B). Most users will likely use the net PTM figure to identify general targets (Figure 5B), but the figure depicting fragments is useful to narrow down complexes of interest based on their unique targeting mechanisms.

**Figure 5:**
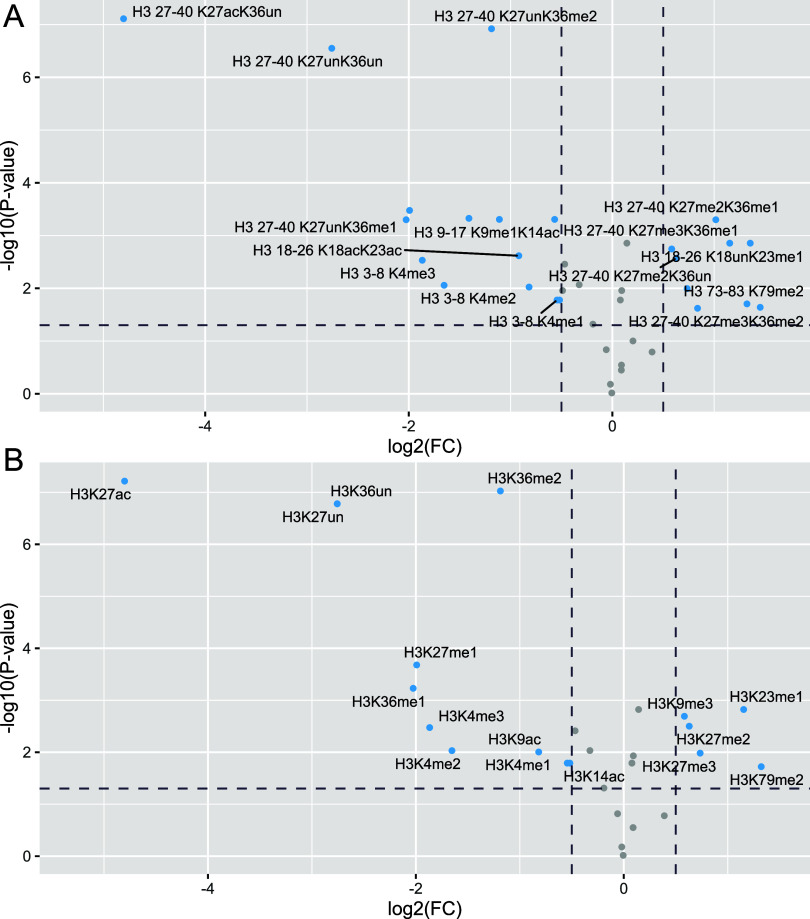
Histone proteome of MCF-7 cells. (A) Fragments as interpreted by mass spectrometry; (B) Net changes of individual PTMs or unmodified residues. In this figure, log2 (Fold Change) represents the log2-transformed fold change of protein expression, initially calculated as MCF-7/average baseline control.

Using the epiproteome atlas data, we generated a comparison of MCF-7 cells to noncancer cell lines to indicate deviations from a healthy state. Here we link differential histone PTMs to frequent findings in breast cancer cells, such as a sharp decrease in H3K27ac. A common pathway of resistance to ER-targeted therapy is downregulation of gene expression through increased DNA methylation at the *ESR1* promoter, generated by low levels of H3K27ac.^78^ This is mitigated through use of histone deacetylase (HDAC) inhibitors, and has shown promise as a combination therapy in treatment-resistant tumors. We also identify upregulation of EZH2-driven H3K27 di- and trimethylation. *ARID1A* mutation is the most common mutation in metastatic breast cancer,^79^ and EZH2 inhibition is known to induce synthetic lethality in other *ARID1A* mutant tumors.^80^

### MPOInt Highlights Overlapping Targets between Estrogen-Responsive Cancers and a Common Comorbidity

To demonstrate use of this tool in different clinical contexts, we examined proteome and phosphoproteome hits from MCF-7 cells in three areas of research, estrogen-responsive breast cancer, hypertension, and ovarian cancer. MCF-7 cells are an estrogen-responsive breast cancer cell line, so we selected identified GDAs for this specific subtype of breast cancer (M18299). Targets of interest may be found in any of the overlapping regions. For brevity, in this section, we examine the center regions containing the overlap between significant genes in the proteome, phosphoproteome, and condition being enriched. This includes proteins regulated on multiple levels, which are most likely to be key influences in disease states.

Figure 6A shows enrichment compared to a clinical data set of genes upregulated in early stage estrogen receptor (ESR1)-positive breast tumors as identified by RNA sequencing.^49^ Here, we identify one hit. The center section contains KDM4B, a histone lysine demethylase that targets histone 3 lysine 9 and reduces chromatin compaction. In tumors, it interacts with both ESR1 and HIF-1α, and its knockdown reduced cell proliferation in hypoxic environments.^81^ In the current analysis, KDM4B expression increased 4-fold in MCF-7 cells with this aggressive phenotype, suggesting it may be a driving factor behind tumor growth and cell proliferation in physiologically relevant conditions.

**Figure 6:**
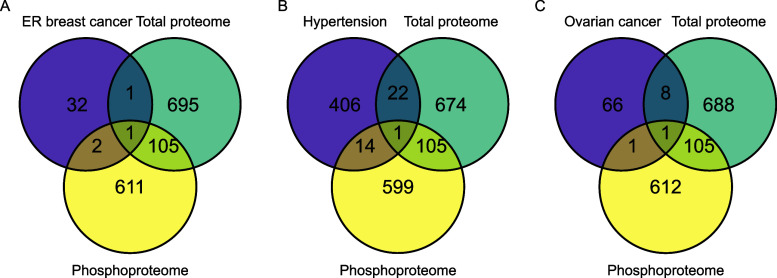
Disease enrichment from 3 areas related to estrogen-responsive breast cancer (ER BC). (A) Enrichment with ER BC; (B) Enrichment with ovarian cancer; (C) Enrichment with hypertension. Purple represents diseases of interest, blue represents the total proteome, and yellow represents the phosphoproteome. Numbers in each section indicate the number of proteins in a category. Full intersection lists are available in Supporting File 1.

We also examined the GDA intersection with hypertension (Figure 6B, HP:0000822), which is the most common comorbidity at diagnosis for patients with ER BC.^82^ The identified molecule in the center section is CTNNB1 (β-catenin). β-catenin is involved in both cancer and hypertension-related pathways.^83,84^ Signaling through the Wnt pathway activates β-catenin translocation to the nucleus, driving cellular proliferation. This tightly regulated pathway is overexpressed in certain cancers, including MCF-7 cells, to promote uncontrolled cell growth.^85^ Hypertension activates Wnt signaling, which also leads to excessive cell growth and fibrosis in vascular endothelia.

Finally, we compared hits from MCF-7 cells with genes of interest in ovarian cancer (Figure 6C, HP:0100615) due to its shared characteristics such as hormone response and treatment choices. Again, here we identify CTNNB1 as the hit shared by all three data sets. β-catenin, as discussed above, is commonly leveraged by tumors, including ovarian cancers.

Interestingly, β-catenin was downregulated in the stem-like condition. This is likely in part due to defective β-catenin accumulation in MCF-7 cells,^86^ which could provide an insight into how tumors redirect growth signals following inactivation of β-catenin by a potential therapy.

### MPOInt Creates a Clear Network Connecting Histone Marks to Epigenetic Regulators Using Three Levels of the Proteome

To complete the integrative analysis, we assess the epigenetic network generated for MCF-7 cells. Again, we present a graphical view of the network to visualize overlap of effector complexes that can influence multiple histone PTMs (Figure 7A). This is particularly relevant due to the higher number of complexes identified within this biological context, many of which share members. Because the histone proteome data examines MCF-7 cells in comparison to noncancerous cells whereas the total and phosphoproteome data compares 3D culture and 2D culture, these findings should be viewed through the lens of MCF-7 cells as a whole rather than being specifically applied to high-stemness MCF-7 cells. We also show the tables generated by the pipeline to describe components in more detail.

**Figure 7:**
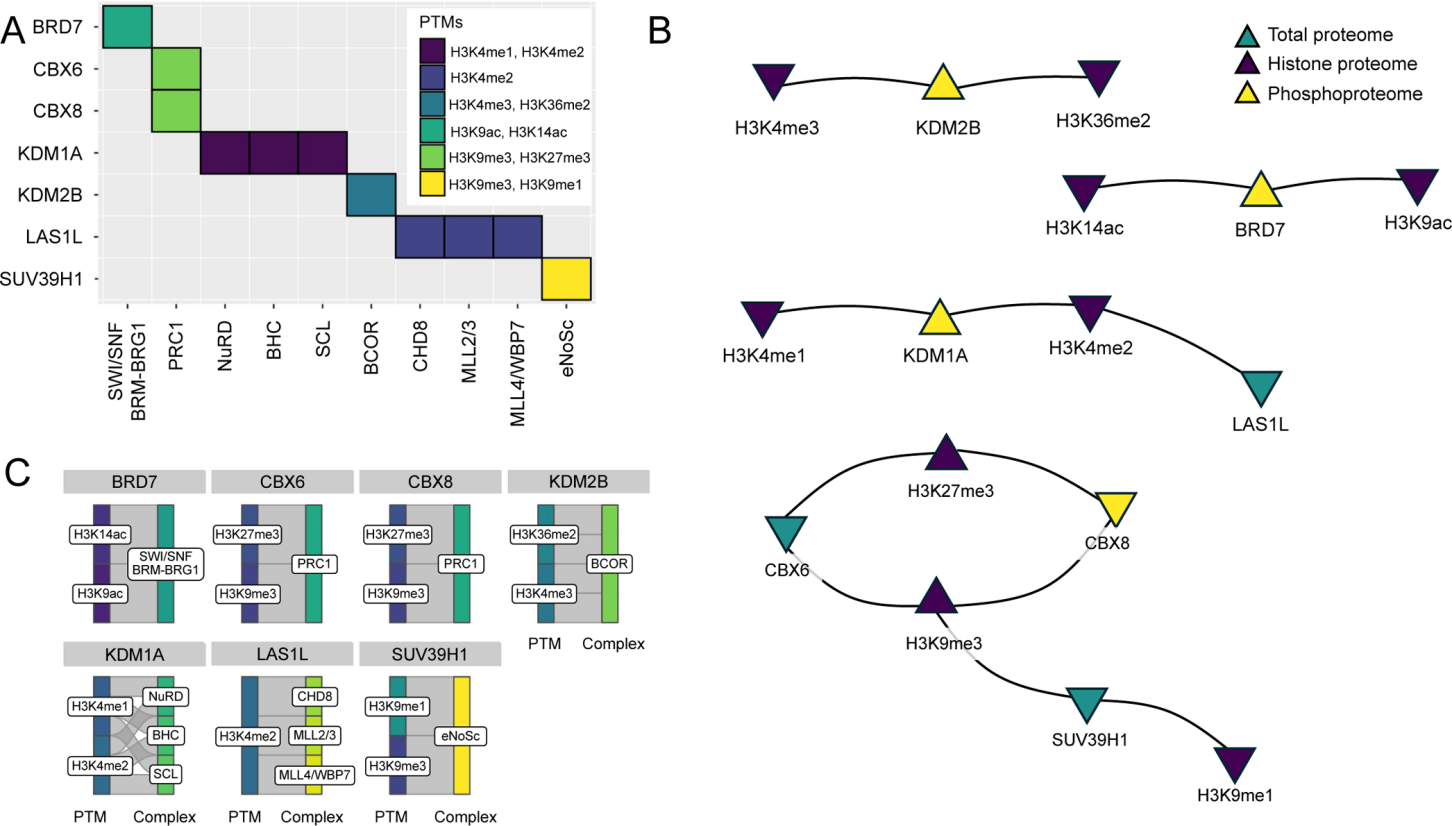
Epigenetic networks from MCF-7 studies. (A) A representation of identified complexes, proteins, and histone PTMs; (B) Histone PTMs assessed in the histone proteome (gray triangles) connected to proteome (red triangles) or phosphoproteome (blue triangles) regulators. The direction of each triangle indicates whether a protein was upregulated or downregulated in the condition of interest; (C) Sankey diagrams showing the connections between PTMs and complexes for each effector.

This network contains a ring connecting H3K27me3 and H3K9me3, both of which are upregulated and linked to CBX6 and CBX8. CBX8 is a known component of the PRC1 complex that contributes to gene silencing, but CBX6 is a more poorly characterized member that has been identified as often downregulated in breast cancer.^87^ Proposed mechanisms connecting PRC1 and PRC1-like complexes describe CBX proteins as gene-specific readers that “read” H3K27me3 at their individual target genes. This enables binding of the full PRC1 complex, promoting chromatin condensation.^88^ Recent findings, however, indicate that CBX proteins may also bind H3K9me3, further increasing the role for PRC1 in gene silencing.^89^

Notably, we also see SUV39H1 downregulated and connected to H3K9me3. SUV39H1 methylates H3K9 in response to cellular energy states and nutrient availability as part of the eNoSC complex^90^ and is known to be overexpressed in breast cancer, especially in aggressive subtypes.^91^ The presence of these five components as highlighted by the MPOInt pipeline indicate a novel and specific mechanism through which breast cancer potentially bypasses nutrient restriction on cell growth.^92–98^

From Table 2, we also see that KDM2B is upregulated in both the total and phosphoproteomes. A brief call to the phosphoproteome data reveals that the identified modification is at position 445 on a serine residue. Using resources such as PhosphoSitePlus, we can further pursue effects of this modification if known and identify other data sets where it is enriched Table 3.

**Table 2:** Proteins Identified in the Network, Their Connected PTMs, and Associated Complexes

symbol	log2(FC) (protein)	source	PTMs	complex
BRD7	3.3516551	Phospho	H3K9ac, H3K14ac	SWI/SNF BRM-BRG1
CBX8	–1.5770182	Phospho	H3K9me3, H3K27me3	PRC1
KDM1A	0.7623012	Phospho	H3K4me1, H3K4me2	NuRD, BHC, SCL
KDM2B	1.7761875	Phospho	H3K4me3, H3K36me2	BCOR
CBX6	–1.3745574	Total	H3K9me3, H3K27me3	PRC1
KDM2B	1.5716614	Total	H3K4me3, H3K36me2	BCOR
LAS1L	–0.3720788	Total	H3K4me2	CHD8, MLL2/3, MLL4/WBP7
SUV39H1	–2.4745292	Total	H3K9me3, H3K9me1	eNoSc

**Table 3:** Histone PTMs Identified in the Network, Their Connected Proteins, and Associated Complexes

PTM	log2(FC) (PTM)	protein	complex
H3K14ac	–0.5186679	BRD7	SWI/SNF BRM-BRG1
H3K27me3	0.7348173	CBX6, CBX8	PRC1
H3K36me2	–1.1871007	KDM2B	BCOR
H3K4me1	–0.5436012	KDM1A	NuRD, BHC, SCL
H3K4me2	–1.6529224	KDM1A, LAS1L	NuRD, BHC, SCL, CHD8, MLL2/3, MLL4/WBP7
H3K4me3	–1.8674603	KDM2B	BCOR
H3K9ac	–0.8182797	BRD7	SWI/SNF BRM-BRG1
H3K9me1	–0.3258271	SUV39H1	eNoSc
H3K9me3	0.5830808	CBX6, CBX8, SUV39H1	PRC1, eNoSc

## Conclusion

We developed a user-friendly pipeline for use in numerous clinical contexts. This pipeline provides quick and customizable visualizations of the overall proteome levels involved, as well as pathway and disease enrichment. This pipeline was validated in two separate diseases by identifying expected targets and linking them to known epigenetic mechanisms associated with each disease.

This study was limited in part by availability of data and open-source resources. Given that there is no unified database for validated biochemical and disease pathways, we chose to source from MSigDB so users have the flexibility to pick from many of their included databases. Primary data of histone proteomics was also limited. We chose to use an atlas of 24 cell lines that examine both cancerous and noncancerous cells, and performed bioinformatic analysis to identify histone PTMs distinct to MCF-7 cells. These analyses provide information for future targeted histone studies.

Excitingly, MPOInt highlights potential new epigenetic targets as described previously, and generates a visualization of hits in an interconnected network. In the studied application of BCG, we identified a potential new mechanism for the effects of BCG-induced anticancer activity through menin scaffolding. Examining MCF-7 cells and cancer stemness also highlighted involvement of CBX proteins and SUV39H1, which may indicate how breast cancer cells continue to promote growth through H3K9-driven epigenetic pathways despite low nutrient availability. It is imperative to create tools that integrate histone proteomics with total and phosphoproteomic data to reveal epigenetic regulatory networks. To our knowledge this is the first tool of its kind. This pipeline provides multiple views of how proteins from each level interact, share targets, and make up common complexes that link seemingly disconnected proteins. Ultimately, MPOInt gives clinically relevant insight into samples including an epigenetic viewpoint by analyzing data from multiple proteome levels, and leverages breadth of existing data that can be explored for mechanistic insight into disease states.

## Supplementary Material



## Data Availability

Data referenced in this manuscript was collected from previous publications and can be retrieved at the following locations. THP-1 total and histone proteome: ProteomeXchange, accession number PXD051187. THP-1 phosphoproteome: PRIDE Database, accession number PXD013171. MCF-7 total and phosphoproteome: ProteomeXchange, accession number PXD046799. MCF-7 histone proteome: Originally uploaded to Tranche, now available on MassIVE under files MSV000078413, MSV000078403, MSV000078401, and MSV000078400.

## Notes

The hits identified in the disease enrichment section of the pipeline for BCG infection and the melanoma genes of interest (Table S1); the hits identified in the disease enrichment section of the pipeline for BCG infection and the RSV genes of interest (Table S2); the proteins identified in the BCG network, their connected PTMs, and associated complexes (Table S3); the histone PTMs identified in the BCG network, their connected proteins, and associated complexes (Table S4); the hits identified in the disease enrichment section of the pipeline for MCF-7 cells and the estrogen-responsive breast cancer genes of interest (Table S5); the hits identified in the disease enrichment section of the pipeline for MCF-7 cells and the hypertension genes of interest (Table S6); the hits identified in the disease enrichment section of the pipeline for MCF-7 cells and the ovarian cancer genes of interest (Table S7) (PDF)
