# Supplementary material for: MPOInt: An Epigenetics-Driven Workflow for Integrative Multi-Level Proteomics
Source: J Proteome Res. 2026 Jun 18;25(7):3304–17. doi: 10.1021/acs.jproteome.5c01076 (PMC13339771; doi:10.1021/acs.jproteome.5c01076)
Supplement: Supplementary file 1 [file pr5c01076_si_001.pdf]

MPOInt: An Epigenetics-Driven Workflow for Integrative Multi-Level Proteomics

*Zoe Schaefer [1], Carleigh Coffin Sokolik [1], and Ivana K. Parker [1,2,3, *]*

1 J. Crayton Pruitt Family Department of Biomedical Engineering, University of Florida, Gainesville, Florida, 32610, USA

2 Emerging Pathogens Institute, University of Florida, Gainesville, Florida, 32610, USA

3 Faculty of African American Studies, University of Florida, Gainesville, Florida, 32610, USA.

* Corresponding author

Email: iparker@bme.ufl.edu

Table S1. The hits identified in the disease enrichment section of the pipeline for BCG infection and the melanoma genes of interest.

Table S2. The hits identified in the disease enrichment section of the pipeline for BCG infection and the RSV genes of interest.

Table S3. The proteins identified in the BCG network, their connected PTMs, and associated complexes.

Table S4. The histone PTMs identified in the BCG network, their connected proteins, and associated complexes.

Table S5. The hits identified in the disease enrichment section of the pipeline for MCF-7 cells and the estrogen-responsive breast cancer genes of interest.

Table S6. The hits identified in the disease enrichment section of the pipeline for MCF-7 cells and the hypertension genes of interest.

Table S7. The hits identified in the disease enrichment section of the pipeline for MCF-7 cells and the ovarian cancer genes of interest.

**Intersection**	**Hits**
Melanoma/Phosphoproteome	ACD, ANAPC1, ATM, BARD1, BRAF, BRCA1, CHEK2, FLCN, MITF, NRAS, PALB2, RAF1, RECQL4, STK11, TERF2IP, ZEB2
Melanoma/Total proteome	-
Total proteome/Phosphoproteome	ACSL1, ALDH2, CMPK2, CNP, DDI2, DERL1, EIF2AK2, EYA3, FKBP8, FTL, GBP1, HELZ2, HMBS, HMOX1, ICAM1, IFI16, IFI44, IFIH1, IFIT1, IFIT2, IFIT3, INPP5F, ISG15, LSM3, MOB1B, MX1, MX2, NAGK, OAS1, OAS3, OASL, PARP14, PKIB, RIGI, RNF213, RRM1, RSAD2, SAMD9, SIRPA, SIRT2, SLFN5, SNRPG, SRSF10, STAT1, STRADA, SULT1A1, TAP2, TAPBP, TUBA1A, TYMP, UBE2D3, UBE2L6, VKORC1
Melanoma/Total proteome/Phosphoproteome	-

Table S1. The hits identified in the disease enrichment section of the pipeline for BCG infection and the melanoma genes of interest.

**Intersection**	**Hits**

RSV/Phosphoproteome	CUL5, EIF2AK2, ELOC, EP300, IFIH1, ISG15, MED1, MED12, RIGI, TRIM25, UBE2L6
RSV/Total proteome	CD14, EIF2AK2, IFIH1, ISG15, OAS2, RIGI, STAT2, TLR2, UBE2L6
Total proteome/Phosphoproteome	ACSL1, ALDH2, CMPK2, CNP, DDI2, DERL1, EIF2AK2, EYA3, FKBP8, FTL, GBP1, HELZ2, HMBS, HMOX1, ICAM1, IFI16, IFI44, IFIH1, IFIT1, IFIT2, IFIT3, INPP5F, ISG15, LSM3, MOB1B, MX1, MX2, NAGK, OAS1, OAS3, OASL, PARP14, PKIB, RIGI, RNF213, RRM1, RSAD2, SAMD9, SIRPA, SIRT2, SLFN5, SNRPG, SRSF10, STAT1, STRADA, SULT1A1, TAP2, TAPBP, TUBA1A, TYMP, UBE2D3, UBE2L6, VKORC1
RSV/Total proteome/Phosphoproteome	EIF2AK2, IFIH1, ISG15, RIGI, UBE2L6

Table S2. The hits identified in the disease enrichment section of the pipeline for BCG infection and the RSV genes of interest.

**Symbol**	**Log2(FC) (protein)**	**Source**	**PTMs**	**Complex**
EZH2	-1.8497518	Phospho	H3K27me1, H3K27me2, H3K27me3	PRC2
KDM2B	-0.8917728	Phospho	H3K4me3, H3K36me2	BCOR
WDR5	0.3132723	Phospho	H3K4me1, H3K4me2, H3K4me3	ATAC, NSL, RING2-L3MBTL2, COMPASS, Menin-associated_HMT, MLL-HCF, CHD8, MLL2/3, COMPASS-like MLL1,2, MLL4/WBP7, COMPASS-like MLL3,4

Table S3. The proteins identified in the BCG network, their connected PTMs, and associated complexes.

PTM	Log2(FC) (PTM)	Gene	Complex
H3K27me1	-0.1610746	EZH2	PRC2
H3K27me2	-0.0831630	EZH2	PRC2
H3K27me3	-0.0336040	EZH2	PRC2
H3K36me2	0.1122873	KDM2B	BCOR
H3K4me1	0.0010933	WDR5	ATAC, NSL, RING2-L3MBTL2, COMPASS, Menin-associated_HMT, MLL-HCF, CHD8, MLL2/3, COMPASS-like MLL1,2, MLL4/WBP7, COMPASS-like MLL3,4
H3K4me2	0.4150375	WDR5	ATAC, NSL, RING2-L3MBTL2, COMPASS, Menin-associated_HMT, MLL-HCF, CHD8, MLL2/3, COMPASS-like MLL1,2, MLL4/WBP7, COMPASS-like MLL3,4
H3K4me3	2.2713020	KDM2B, WDR5	BCOR, ATAC, NSL, RING2-L3MBTL2, COMPASS, Menin-associated_HMT, MLL-HCF, CHD8, MLL2/3, COMPASS-like MLL1,2, MLL4/WBP7, COMPASS-like MLL3,4

Table S4. The histone PTMs identified in the BCG network, their connected proteins, and associated complexes.

Intersection	Hits
ER BC/Phosphoproteome	AHNAK, KDM4B, MAGED2
ER BC/Total proteome	ANXA9, KDM4B

Total proteome/Phosphoproteome	AHNAK2, AJUBA, AKAP13, ALDOC, ANKRD17, ARHGAP29, ARHGEF18, ASH2L, ASPSCR1, ATP2B1, BAG3, BCKDHA, BIN1, BRSK2, BTRC, BUB1, CAMKK1, CCZ1, CD83, CDC20, CDC25C, CDK18, CHML, CLASP2, CTNNB1, CTTNBP2, DAB2, DAG1, DDB2, DDX24, DIAPH3, EHBP1L1, EIF4EBP2, EIF4G2, ELAVL1, FARP2, FERMT2, FNTB, IRS2, IVNS1ABP, JAG2, JUNB, KCTD5, KDM3A, KDM4A, KDM4B, KIF20A, KLHDC10, LARP4, LPIN3, LRRC41, MLXIP, NCAPD2, NEMF, NFKB2, NOP2, NUFIP1, NUFIP2, NYAP2, OSER1, PARP1, PHLDB1, PKP3, PPA2, PPP1R15B, PPP2R3A, PRR11, PRRC2B, PTGFRN, RABGGTB, RAD54B, RBM14, RBM4, RBM47, RNF25, RPL27A, RPL31, RPS10, RPUSD2, SAMHD1, SHMT2, SKA3, SKP2, SLC1A5, SLC6A15, STBD1, TACC1, TBL1XR1, TCF3, TJP3, TNRC6B, TP53RK, TRAF7, TTF2, TTK, UBAP2, UHRF1, USP6NL, VEZF1, VRK3, XRN2, YBX1, YBX3, YTHDF2, ZFAND5, ZNF800
ER BC/Total proteome/Phosphoproteome	KDM4B

Table S5. The hits identified in the disease enrichment section of the pipeline for MCF-7 cells and the estrogen-responsive breast cancer genes of interest.

**Intersection**	**Hits**
Hypertension/Phosphoproteome	CTNNB1, CTR9, ERCC6, INF2, JMJD1C, KDM1A, LDLRAP1, LIMK1, MEF2A, MTRR, MUC1, NOTCH2, REST, SLC20A2, SMARCAL1
Hypertension/Total proteome	ALMS1, ARVCF, BAZ1B, BICC1, CCND1, CDKN1B, CEP83, CTNNB1, ENG, EXT2, FDXR, H4C3, H6PD, IMPDH2, KIAA0319L,

	LOX, MAX, MECP2, PRNP, SDHA, TGFBR2, TRIM32, VANGL1
Total proteome/Phosphoproteome	AHNAK2, AJUBA, AKAP13, ALDOC, ANKRD17, ARHGAP29, ARHGEF18, ASH2L, ASPSCR1, ATP2B1, BAG3, BCKDHA, BIN1, BRSK2, BTRC, BUB1, CAMKK1, CCZ1, CD83, CDC20, CDC25C, CDK18, CHML, CLASP2, CTNNB1, CTTNBP2, DAB2, DAG1, DDB2, DDX24, DIAPH3, EHBP1L1, EIF4EBP2, EIF4G2, ELAVL1, FARP2, FERMT2, FNTB, IRS2, IVNS1ABP, JAG2, JUNB, KCTD5, KDM3A, KDM4A, KDM4B, KIF20A, KLHDC10, LARP4, LPIN3, LRRC41, MLXIP, NCAPD2, NEMF, NFKB2, NOP2, NUFIP1, NUFIP2, NYAP2, OSER1, PARP1, PHLDB1, PKP3, PPA2, PPP1R15B, PPP2R3A, PRR11, PRRC2B, PTGFRN, RABGGTB, RAD54B, RBM14, RBM4, RBM47, RNF25, RPL27A, RPL31, RPS10, RPUSD2, SAMHD1, SHMT2, SKA3, SKP2, SLC1A5, SLC6A15, STBD1, TACC1, TBL1XR1, TCF3, TJP3, TNRC6B, TP53RK, TRAF7, TTF2, TTK, UBAP2, UHRF1, USP6NL, VEZF1, VRK3, XRN2, YBX1, YBX3, YTHDF2, ZFAND5, ZNF800
Hypertension/Total proteome/Phosphoproteome	CTNNB1

Table S6. The hits identified in the disease enrichment section of the pipeline for MCF-7 cells and the hypertension genes of interest.

**Intersection**	**Hits**
Ovarian cancer/Phosphoproteome	CTNNB1, MBD4
Ovarian cancer/Total proteome	CTNNB1, DICER1, IDH2, KEAP1, MAP3K1, PALLD, PTEN, RABL3, TGFBR2

Total proteome/Phosphoproteome	AHNAK2, AJUBA, AKAP13, ALDOC, ANKRD17, ARHGAP29, ARHGEF18, ASH2L, ASPSCR1, ATP2B1, BAG3, BCKDHA, BIN1, BRSK2, BTRC, BUB1, CAMKK1, CCZ1, CD83, CDC20, CDC25C, CDK18, CHML, CLASP2, CTNNB1, CTTNBP2, DAB2, DAG1, DDB2, DDX24, DIAPH3, EHBP1L1, EIF4EBP2, EIF4G2, ELAVL1, FARP2, FERMT2, FNTB, IRS2, IVNS1ABP, JAG2, JUNB, KCTD5, KDM3A, KDM4A, KDM4B, KIF20A, KLHDC10, LARP4, LPIN3, LRRC41, MLXIP, NCAPD2, NEMF, NFKB2, NOP2, NUFIP1, NUFIP2, NYAP2, OSER1, PARP1, PHLDB1, PKP3, PPA2, PPP1R15B, PPP2R3A, PRR11, PRRC2B, PTGFRN, RABGGTB, RAD54B, RBM14, RBM4, RBM47, RNF25, RPL27A, RPL31, RPS10, RPUSD2, SAMHD1, SHMT2, SKA3, SKP2, SLC1A5, SLC6A15, STBD1, TACC1, TBL1XR1, TCF3, TJP3, TNRC6B, TP53RK, TRAF7, TTF2, TTK, UBAP2, UHRF1, USP6NL, VEZF1, VRK3, XRN2, YBX1, YBX3, YTHDF2, ZFAND5, ZNF800
Ovarian cancer/Total proteome/Phosphoproteome	CTNNB1

Table S7. The hits identified in the disease enrichment section of the pipeline for MCF-7 cells and the ovarian cancer genes of interest.
